# Draft Genome Sequences of Two Isolates of the Yeast Kazachstania servazzii Recovered from Soil in Ireland

**DOI:** 10.1128/MRA.01257-19

**Published:** 2019-10-31

**Authors:** Lynne Faherty, Clifton Lewis, Matt McElheron, Niall Garvey, Róisín Duggan, Ben Shovlin, Tadhg Ó Cróinín, Kevin P. Byrne, Caoimhe E. O’Brien, Kenneth H. Wolfe, Geraldine Butler

**Affiliations:** a School of Biomolecular and Biomedical Science, Conway Institute, University College Dublin, Dublin, Ireland; b School of Medicine, Conway Institute, University College Dublin, Dublin, Ireland; Vanderbilt University

## Abstract

We sequenced two isolates of Kazachstania servazzii, UCD13 and UCD335, from soil in Ireland. Heterozygosity in these diploid genomes differs 19-fold between the two strains. Most currently available *K. servazzii* genome sequences come from Korean kimchi isolates, so our data will facilitate analysis of diversity in this species.

## ANNOUNCEMENT

Kazachstania servazzii is an ascomycete yeast in the family Saccharomycetaceae. It was formerly called Saccharomyces servazzii. The type strain was isolated from soil in Finland in 1967 (1). *K. servazzii* is not pathogenic. It contributes to enhanced flavor through isoamyl alcohol production in fermented foods, including Camembert cheese, kimchi, and sourdough (2–4). It has also been implicated in changing flavor profiles through ethyl decanoate production during Chinese liquor fermentation (5). Conversely, *K. servazzii* is associated with food spoilage, such as swelling of pizza packaging due to gas production (3) and formation of undesirable white yeast colonies on kimchi (6).

Genome survey sequencing of the *K. servazzii* type strain was reported in 2000 (7), and the whole-genome sequence of an isolate from kimchi (strain CBA6004) was recently reported (6). The genome sequence of a second kimchi isolate is also available (NCBI BioProject accession number PRJNA390859).

Strain UCD13 was isolated in 2017 from a soil sample from woodland in County Tipperary, Ireland (global positioning system [GPS] coordinates, 52.52355, −8.13510). UCD335 was isolated in 2018 from soil in a Dublin, Ireland, garden (GPS coordinates, 53.28961, −6.28822). The internal transcribed spacer (ITS) region of rRNA genes was amplified using the primers ITS1 and ITS4 (8) and sequenced using Sanger technology (GenBank accession numbers MN540706 and MN540707).

The yeasts were grown in yeast extract-peptone-dextrose broth. Total genomic DNA was isolated by phenol-chloroform extraction and purified using a Zymo Research DNA Clean & Concentrator-25 kit (UCD13) or a Qiagen QiaAMP DNA minikit (UCD335). Libraries were generated and sequenced by BGI Tech Solutions (Hong Kong). Genomic DNA (1 μg) was fragmented using Covaris, purified with an AxyPrep Mag PCR cleanup kit, and end repaired; A tails were added by using an A-tailing mix and incubating at 37°C for 30 min. Illumina adapters were ligated by incubating at 16°C for 16 h. Insert sizes of ∼800 bp were selected, and 150 bases were sequenced from each end with an Illumina HiSeq 4000 platform, yielding 6.4 million spots for UCD13 and 9.6 million spots for UCD335. Scaffolds were assembled using Redundans v0.14a (9). All parameters used for sequence assembly and analysis are available at https://doi.org/10.6084/m9.figshare.9963923.

QUAST v4.6.1 (10) showed that the UCD13 assembly totaled 12.0 Mb, with a G+C content of 34.2%. The *N*_50_ value was 169,222 bp, and the *L*_50_ value was 22. The UCD335 assembly was 11.76 Mb, the *N*_50_ value was 281,905 bp, and the *L*_50_ value was 14. The mitochondrial genomes were identified by tBLASTn searches using S. cerevisiae mitochondrial proteins as queries and have GenBank accession numbers VWSC01000085 and VWSB01000061.

Variant analysis used the Burrows-Wheeler Aligner MEM (BWA-MEM) algorithm v0.7.12-r1039 (11), SAMtools v1.1.19 (12), and the Genome Analysis Toolkit (GATK) v4.0.1.2 (13). Variants were filtered by removing clusters (5 variants within 20 bp) that were assumed to result from poor read alignment and by applying GATK filters (QualByDepth [QD], <2.0; Mapping Quality [MQ], <40.0; FisherStrand [FS], >60.0; StrandOddsRatio [SOR], >3.0; MappingQualityRankSumTest [MQRankSum], less than –12.5; and ReadPosRankSumTest [ReadPosRankSum], less than –8.0). There were approximately 73,500 heterozygous single nucleotide polymorphisms (SNPs) and 9,400 insertions/deletions (indels) in UCD13. In contrast, UCD335 had only approximately 3,750 heterozygous SNPs and 960 indels. Analysis of biallelic SNP frequency distribution indicated that UCD13 has a heterozygous diploid genome (i.e., a 50/50 distribution of the reference and nonreference alleles was observed). The level of heterozygosity in UCD13 (6.1 SNPs per kb) is slightly less than the highest level seen in a survey of 1,011 S. cerevisiae isolates (14).

### Data availability.

This whole-genome shotgun project has been deposited at DDBJ/ENA/GenBank under the accession numbers VWSB00000000 and VWSC00000000 (UCD335 and UCD13, respectively), and the raw reads are at the Sequence Read Archive under accession numbers SRX6818742 and SRX6818741. The ITS sequences have accession numbers MN540706 and MN540707, and the mitochondrial genomes are at VWSB01000061 and VWSC01000085. The data are also available under BioProject accession number PRJNA564535.
